# Therapeutics in Osteoarthritis Based on an Understanding of Its Molecular Pathogenesis

**DOI:** 10.3390/ijms19030674

**Published:** 2018-02-27

**Authors:** Ju-Ryoung Kim, Jong Jin Yoo, Hyun Ah Kim

**Affiliations:** 1 Rheumatology Division, Department of Internal Medicine, Hallym University Sacred Heart Hospital, 896, Pyongchondong, Dongan-gu, Anyang, Kyunggi-do 431-070, Korea; jurykim75@gmail.com; 2 Department of Internal Medicine, Kangdong Sacred Heart Hospital, Seoul 05355, Korea; 99jjyoo@hanmail.net

**Keywords:** osteoarthritis (OA), articular cartilage, molecular pathology, therapeutics

## Abstract

Osteoarthritis (OA) is the most prevalent joint disease in older people and is characterized by the progressive destruction of articular cartilage, synovial inflammation, changes in subchondral bone and peri-articular muscle, and pain. Because our understanding of the aetiopathogenesis of OA remains incomplete, we haven’t discovered a cure for OA yet. This review appraises novel therapeutics based on recent progress in our understanding of the molecular pathogenesis of OA, including pro-inflammatory and pro-catabolic mediators and the relevant signalling mechanisms. The changes in subchondral bone and peri-articular muscle accompanying cartilage damage are also reviewed.

## 1. Introduction

Osteoarthritis (OA) is the most prevalent joint disease in older people and is characterized by the progressive destruction of articular cartilage, synovial inflammation, changes in subchondral bone and peri-articular muscle, and pain. The progression of OA is usually slow. Nevertheless, it ultimately leads to joint disability because of the poor repair capacity of cartilage [1,2]. Although various risk factors associated with OA are known, including genetic predisposition, aging, obesity, mechanical stress, and traumatic joint injury, the exact aetiology of OA remains largely unknown [3,4] and we have not discovered a cure for it. Therefore, it is important to appreciate the multi-factorial pathology of OA.

This review appraises novel therapeutics based on recent progress in our understanding of the molecular pathogenesis of OA.

## 2. Molecular Pathology of Cartilage Destruction

In healthy cartilage, chondrocytes respond to their microenvironment to maintain a delicate balance between synthesis and degradation of the extracellular matrix (ECM), the major components of which are type II collagen and aggrecan [5]. When the normal physiological mechanism that maintains the matrix equilibrium fails, ECM components are lost, expanded chondrocytes cluster in the depleted regions, an oxidative state is induced in the stressed cellular environment, and ultimately chondrocyte apoptosis occurs [6,7]. Failure of matrix equilibrium is due to the increased expression of matrix-degrading enzymes [8], inhibition of matrix synthesis [9], and excessive production of pro-inflammatory mediators, including cytokines, chemokines, and matrix degradation products [9]. Subchondral bone changes lead to osteophyte formation and sclerosis; loosening and weakness of the peri-articular muscles accompanies the articular cartilage destruction [10,11].

### 2.1. Pro-Inflammatory Cytokines

Osteoarthritis was once considered the prototypical non-inflammatory arthropathy distinct from rheumatoid arthritis (RA), a systemic autoimmune disease characterized by chronic inflammation. However, current research has demonstrated that inflammation is one of the key factors leading to the destruction of cartilage in OA. In the OA synovium, inflammatory cell infiltration is frequently observed, sometimes to a similar degree to that seen in RA. However, it is unclear whether this inflammation is the cause or consequence of cartilage destruction. Among inflammatory mediators, the role of cytokines has been studied the most, and many cytokines have been found in OA joints, in correlation with the severity of inflammation, and these play various roles in disrupting the balance of catabolic and anabolic activity in joint tissues [12]. IL-1β, IL-6, and TNF-α cytokines play the most important roles in pathogenesis and disease severity of OA [13], while IL-15, IL-17, IL-18, IL-21 [14], and chemokines and their receptors, such as MCP-1/CCL2, IL-8/CXCL8, and GRO-α/CXCL1, have also been implicated [15]. IL-1β is produced by several cell types in joints, including chondrocytes, immune cells infiltrating the synovium, osteoblasts, adipocytes, and synoviocytes; IL-1β expression is elevated in OA synovial fluid and membranes during the development of OA [16]. IL-1β strongly induces the expression and release of proteolytic enzymes, such as matrix metalloproteinases (MMPs) and aggrecanases, and suppresses the expression of ECM components, including type II collagen and aggrecan [17,18]. It also acts synergistically with other cytokines, IL-6 and chemokines including IL-8, MCP-1, and CCL5, to further increase inflammation [14]. Nevertheless, the elimination of IL-1β in a mouse model of traumatic joint injury aggravated OA, indicating a more complex role for this cytokine in maintaining cartilage metabolism [19]. TNF-α is also elevated in OA joint tissues and synovial fluid compared with healthy individuals [12]. Expression of the p55 TNF-α receptor has been localized in cells at sites of focal loss of cartilage proteoglycans in human OA [20]. TNF-α suppresses the synthesis of proteoglycan and type II collagen in chondrocytes [21] and stimulates pro-inflammatory and pro-catabolic mediators such as MMP-1, -3, and -13, IL-6, IL-8, and chemokines such as MCP-1 and CCL5 [22]. Furthermore, TNF-α promotes the production of nitric oxide (NO), a potent catabolic and pro-apoptotic mediator, in the synovial tissue, while blockade of the TNF-α receptor results in the inhibition of NO production in human cartilage tissue [23].

### 2.2. Pro-Catabolic Factors

Biomechanical stress, genetic factors, and inflammation contribute to the development of OA by interfering with metabolic responses in chondrocytes that maintain matrix integrity [24]. A series of pro-catabolic and anti-anabolic factors have been identified in the destruction of articular cartilage in OA. In the early phase, anabolic activity is increased, but this response fails to repair the cartilage due to both quantitative and qualitative insufficiency [25], as well as the intrinsic limitation of cartilage repair. During the development of OA, catabolic activity is triggered by pro-inflammatory cytokines, including IL-1β, IL-6, IL-17, and TNF-α. Elevated inducible nitric oxide synthase (iNOS) levels in OA chondrocytes result in an excess of NO, which suppresses proteoglycan and collagen synthesis in chondrocytes [26] and mediates the induction of matrix-degrading MMPs by accelerating the catabolic cascade induced by IL-1β or TNF-α. Chondrocyte-derived MMPs are the main enzymes involved in the breakdown of cartilage collagens and proteoglycans, while pro-inflammatory cytokines up-regulate the expression of MMPs via sequential activation of the catabolic cascade. MMP-13 effectively degrades type II collagen and MMP-13 knockout (KO) mice are protected from cartilage destruction in a surgical OA model [27], while constitutive expression of MMP-13 in transgenic mice induced spontaneous cartilage degradation [28]. MMP activity is partially inhibited by the tissue inhibitors of MMPs (TIMPs), whose synthesis is low compared with MMP production in OA cartilage. The importance of TIMPs in the development of cartilage degradation was demonstrated by elevated serum levels of type II collagen cleavage products in TIMP-3 KO mice [29]. Aggrecan is a large proteoglycan and a critical component of the ECM, along with type II collagen. It is degraded by aggrecanases, particularly ADAMTS-4 and ADAMTS-5, both of which are expressed in human OA cartilage. IL-1 and TNF-α up-regulate ADAMTS-4, but not ADAMTS-5, in human synovial cells [30]. In contrast to humans, in mice, IL-1 elevates ADAMTS-5 expression [31] and ADAMTS-5 KO mice are resistant to cartilage erosion in a surgical OA model, suggesting that ADAMTS-5 is the predominant aggrecanase responsible for the development of OA in mice [32].

### 2.3. Transcription Factors

The role of signalling pathways, including the Notch, HIF-2 α, and NF-κB pathways, has been studied in the pathogenesis of OA. The increased expression and activation of Notch signalling components, such as Notch-1 and 2 receptors, Notch ligand, Jag1 and the downstream effector Hes1, have been identified in human OA and a mouse surgical OA model [33,34]. Although several downstream effectors mediate the effect of Notch signalling, including Hes1, 5, 7, Hey 1, 2, and HeyL, only Hes1 expression has been demonstrated in articular chondrocytes and its overexpression induced MMP 13 expression [34]. Furthermore, Hes1 KO mice were resistant to cartilage destruction and showed decreased MMP13 expression in a mouse surgical OA model [34], suggesting that Hes1 mediates the catabolic effect of Notch signalling. However, the role of the Notch signalling pathway in the homeostasis of articular cartilage and OA remains controversial, since transient Notch activation promotes ECM synthesis and helps to maintain articular cartilage under physiological conditions [35]. Articular cartilage is avascular and maintained in a low-oxygen environment with an oxygen gradient in cartilage that ranges from around 6% at the joint surface to 1% in the deep layers [36]. The hypoxia-inducible factor (HIF) protein family permits chondrocytes to adapt to hypoxic conditions. In mice, cartilage-specific HIF-1α deletion leads to articular chondrocyte death, whereas inhibition of HIF-1α degradation increases accumulation of the ECM [37,38]. Mice heterozygous for HIF-2α (or endothelial PAS domain-containing protein 1, EPAS-1) are resistant to developing OA [39]. HIF-2α induces numerous catabolic mediators, including MMPs (MMP1, MMP3, MMP9, MMP12 and MMP13), ADAMTS4, nitric oxide synthase-2 (NOS2), and prostaglandin-endoperoxide synthase-2 (PTGS2) [40]. Therefore, HIF-2 α is believed to be a central transactivator that causes cartilage destruction by regulating key catabolic genes. NF-κB is activated by inflammatory cytokines, elicits the secretion of many degradative enzymes, including MMPs and ADAMTSs, and suppresses ECM synthesis molecules such as Sox9, thereby down-regulating the ECM components type II collagen and aggrecan [30]. NF-κB also acts in a positive feedback loop to augment the catabolic process by stimulating NF-κB-mediated inflammatory cytokines, such as TNF-α, IL-1β, and IL-6, and the chemokine IL-8, and receptor activator of NF-κB (RANK) ligand (RANKL), leading to ECM breakdown and subsequent cartilage destruction [41]. In support of this, knockdown of NF-κB signalling by siRNA has been shown to inhibit cartilage degradation in a mouse model of OA [42]. Adenoviral gene delivery of the NF-κB inhibitor IκBa protects against cartilage loss by suppressing the expression of several MMPs and aggrecanases [43,44], which indicates that the NF-κB signalling plays a central role in degenerative cartilage disease. A recent study showed that the Zn^2+^ transporter ZIP8 was upregulated in OA cartilage of humans and mice, and the ZIP8-mediated Zn^2+^ influx upregulated the expression of matrix-degrading enzymes in chondrocytes [45]. Metal-regulatory transcription factor-1 (MTF1) was identified as an essential transcription factor in the mediation of Zn^2+^/ZIP8-induced catabolic factor expression, and Ad-Mtf1 injection in mice caused osteophyte formation and subchondral bone sclerosis with more severe cartilage destruction, suggesting that the zinc-ZIP8-MTF1 axis is a major catabolic regulator of the pathogenesis of OA.

### 2.4. Inherent Changes in Chondrocytes: Senescence, Apoptosis, Autophagy

OA is a disease prevalent in advanced age, characterized by reduction of repair mechanism of cell damage. Cellular senescence is considered a signal transduction process that results in cells entering a stable state of growth arrest, and ultimately results in the loss of cellular replication [46]. Senescent cells are identified by a constellation of characteristics, such as absence of proliferation markers, increase in cell volume, an activated DNA damage response and expression of senescence-associated β-galactosidase (SA-Bgal) [47]. In addition to reduction of tissue regenerative potential, senescent cells chronically secrete proteases and pro-inflammatory mediators, (senescence-associated secretory phenotype (SASP)), which may perturb tissue structure and create a tissue microenvironment promoting proliferation of neoplastic cells [48]. Aged chondrocytes display a number of characteristics detrimental to cartilage integrity: they are more susceptible to cell death induced by an NO donor and less responsive to growth factors, such as insulin-like growth factor(IGF)-1 and osteogenic protein-1 [49]. When compared to cells isolated from young donors, chondrocytes from older adults secrete more MMP-13, IL-1 and IL-7, which are characteristics of SASP [50,51]. Application of cell senescence regulation in the treatment of OA is at its early phase. A recent study using p16-3MR transgenic mouse, which allows selective removal of senescent cells, showed that selective elimination of these cells attenuated the development of cartilage destruction after anterior cruciate ligament transection (ACLT), reduced pain and increased cartilage development [52]. Intra-articular injection of a senolytic molecule that selectively kills senescent chondrocytes led to attenuation of articular cartilage degeneration and amelioration of pain in ACLT-induced OA in C57BL mice as well, suggesting that regulation of senescence may serve as a therapeutic target for OA.

Apoptosis is a highly regulated process of cell death involving specific sets of intracellular signalling pathway. During the late phase of OA, cartilage becomes hypocellular with numerous empty lacunae, and the rate of apoptotic chondrocytes has been reported as high as 20% [53]. While it is intuitive that the death of chondrocytes, the only cell residing in cartilage, would result in the failure to appropriately maintain the structure of articular cartilage, a high rate of apoptosis in cartilage would result in matrix degradation within a short period of time, which is not compatible with the chronic course of OA [54]. Chondrocyte apoptosis may lead to reduction of ECM, and decrease of ECM may in turn result in further chondrocyte apoptosis because of the loss of matrix–cell interaction and anchorage dependence, eventually causing a viscous cycle [55]. Attempts at employing apoptosis modulators for treatment of OA has been hampered by potential harmful effects such as cancer, and inhibitors of apoptosis have been tested mostly in pre-clinical studies. For example, caspase inhibitor, the key regulator of apoptosis, was used in canine and rabbit model of OA, and found to suppress chondrocyte apoptosis as well as cartilage degradation [56,57,58]. Recently, autophagy, an adaptive response to protect cells from stresses, has been gaining interest as a regulatory mechanism of chondrocyte apoptosis [59]. Autophagy is induced by a variety of stimuli, including metabolic stress and hypoxia, and regulates catabolic processes of energy recycling in eukaryotic cells [60]. Autophagy has been shown to have a complex cross-talk with apoptosis and is induced by common upstream signals. Previous reports have shown that autophagy is decreased in OA cartilage and in an animal OA model, and autophagy activation protected chondrocytes from death, suggesting that autophagy is protective in cartilage degradation [61]. Chondrocyte autophagy is activated by hypoxia inducible factor-1 (HIF-1)-dependent AMP activated protein kinase (AMPK) and mammalian target of rapamycin (mTOR), while HIF-2α inhibits HIF-1α-induced autophagy [62] [63]. Resveratrol (3,4′,5-trihydroxystilbene) is an active food ingredient from grapes and peanuts, which was found to extend life span in nematodes, and ameliorate the fitness of human cells undergoing metabolic stress. Resveratrol was found to prevent cartilage destruction in a mouse model of OA by activating Sirtuin 1 and suppressing the expression of HIF-2α and catabolic mediators such as MMP 13 and ADAMTS5 [64]. Interestingly, a recent study showed that intra-articular injection of resveratrol led to an increase in autophagy markers such as Unc-51-like kinase1, Beclin1, and microtubule-associated protein light chain 3, and delayed articular cartilage degradation in DMM-induced OA. These results indicate that autophagy regulators such as resveratrol may be therapeutic targets for OA.

## 3. Molecular Pathology of Bone and Peri-Articular Muscle

Bone remodelling is thought to play an important role in the pathophysiology of OA and numerous studies have revealed that changes in cartilage and bone are tightly coupled to the progression of OA. The subchondral bone underneath the articular cartilage is organized into the subchondral cortical plate and a layer of cancellous bone [65]. In the early stage of OA, subchondral bone remodelling increases. Cellular signalling for microdamage repair, stimulation of vascular invasion by angiogenic factors, and the disruption of cartilage crosstalk via pathological microcracks are responsible for this increased subchondral bone remodelling [66]. In the late stage of OA, bone remodelling decreases and subchondral bone sclerosis increases, along with thickening of the cortical plate. Despite this thickening, the stiffness of the subchondral bone decreases due to decreased mineralization [67]. In addition, new bone formation characterized by osteophytes [68] and increased osteoblastic activity, dominate the late phase of OA [69], and subchondral bone cysts and bone marrow lesions are present [70].

In addition to cartilage, subchondral bone is also exposed to catabolic factors, such as MMPs and ADAMTSs, chemokines, and inflammatory cytokines secreted by hypertrophic chondrocytes, and these factors have been implicated in the altered biochemical and functional abilities of osteoblasts [71]. For instance, osteoblasts switch from the normal phenotype to a sclerotic phenotype on exposure to IL-6 in combination with other cytokines, such as IL-1β [71]. The penetration of vessels into articular cartilage exposes chondrocytes to cytokines and growth factors from subchondral bone, such as vascular endothelial growth factor (VEGF), nerve growth factor (NGF), IL-1, IL-6, hepatocyte growth factor (HGF), and insulin-like growth factor (IGF)-1 [69].

Several signalling pathways have been implicated in the changes in subchondral bone that contribute to the progression of OA, including Wnt, TGF-β/BMP, and MAPK signalling. Of these, the Wnt/β-catenin signalling pathway has emerged as a key regulator of bone remodelling. For example, activation of Wnt signalling was observed in osteocytes in subchondral bone, and altered Wnt activation, either by knockdown of Wnt antagonists or overexpression of β-catenin, resulted in increased bone formation in an animal model, leading to thicker and stiffer bones [72,73,74]. More recently, Zhong et al. [75] provided a possible cross-talk between IL-1b and Wnt signalling in OA. Their findings revealed that IL-1b decreased the expression of Wnt antagonist, Dickkopf-1 (DKK1) and Frizzled related protein (FRZB), through upregulation of iNOS/NO, thereby activating the transcription of WNT target genes in human chondrocyte [75].

In addition to cartilage degradation and abnormal subchondral bone remodelling, pathological peri-articular muscle weakness appears in OA. There is increasing evidence that the consequences of OA, such as pain, joint instability, maladaptive postures, and defective neuromuscular communication, are associated with decreased lower limb muscle strength or function [76]. However, it remains unclear whether this change in peri-articular muscle is responsible for the diseased onset and progression, or is a consequence of the degenerative joint. Although OA is defined as a loss of articular cartilage within the joint, muscle impairment associated with the OA may be the primary underlying cause of the functional limitations [77], and muscle dysfunction may actually lead to a further increase in cartilage deterioration. The quadriceps, hamstrings, and hip muscles are all significantly impaired in patients with knee OA, and the quadriceps, which is involved in functional tasks such as standing up from a chair, going up and down stairs, and level surface walking, is a central determinant of physical function in patients with knee OA [78,79]. Greater quadriceps strength is associated with protection from cartilage loss in the lateral compartment of the patellofemoral joint [80]. The muscle mass loss induced by botulinum type-A toxin injections in rabbits led to cartilage degradation four weeks after injury, suggesting that muscle weakness can cause degenerative joint disease [81]. Proteoglycan loss occurs in the cartilage of mdx/utrophin^−/−^ mice, which lack both dystrophin and utrophin, two important skeletal muscle structural proteins, demonstrating that skeletal muscle plays a crucial role in maintaining cartilage integrity [82]. A surgically induced OA mouse model exhibited impaired muscle function, with changes in twitch and tetanic force in the tibialis anterior muscle [83]. Although the link between the molecular regulation of peri-articular muscle function and knee OA is still under investigation, inflammation in muscles surrounding the knee may cause muscle weakness in knee OA. Inflammatory mediators, such as monocyte chemotactic protein 1 (MCP-1), p65 NF-κB, and JNK-1, are increased in the muscles of patients with knee OA, and were found to correlate with altered knee function, slower gait velocity, and greater physical disability [84,85]. These markers also upregulate pro-inflammatory cytokines, including TNF-α, IL-1β, Il-6, and IL-8, and atrogin-1, a muscle-specific atrophy-related E3 ubiquitin ligase [86,87]. Most studies have focused on the inflammatory responses within the synovium and articular chondrocytes; however, these findings suggest that the inflammatory response in patients with knee OA affects peri-articular tissues, such as subchondral bone and skeletal muscle.

## 4. Novel Therapeutics Based on the Molecular Pathogenesis of OA

### 4.1. Anti-Inflammatory and Cytokine Blocker

#### 4.1.1. Anti TNF-α Therapies

TNF-α blockers are very effective in inflammatory joint diseases such as RA, and TNF-α plays a considerable role in the pathogenesis of OA. However, their effects on OA disease modification have not been proven clinically. Adalimumab is a human monoclonal antibody bioengineered to bind to TNF-α and prevent receptor binding [88]. A 12-month randomised, double-blind, placebo-controlled trial evaluated the efficacy of adalimumab (subcutaneously 40 mg every two weeks) in 60 patients with erosive hand OA [89]. Progression from palpable soft tissue swelling to joint damage decreased ten-fold in the adalimumab group compared with the placebo group. Although fewer adalimumab-treated patients developed erosive OA in their interphalangeal joints than the placebo arm (26.7% vs. 40%), the difference was not significant. In a randomised, double-blind, placebo-controlled, multicentre study, 85 patients with hand OA who were non-responders to analgesics and non-steroidal anti-inflammatory drugs (NSAIDs) received adalimumab (40 mg) or placebo subcutaneously every 15 days, and adalimumab was not superior to placebo for alleviating pain [90]. Infliximab has been suggested to reduce the secondary hand OA in patients with RA [91].

An open-label randomized controlled study involving 56 patients with moderate to severe knee OA received an intraarticular injection either 10 mg adalimumab, or 25 mg hyaluronic acid [92]. The decrease in the pain visual analog scale (VAS) score, Western Ontario and McMaster Universities Arthritis Index (WOMAC) score, Patient Global Assessment score, and Physician Global Assessment score from baseline to week 4 were greater in the adalimumab than hyaluronic acid group. There was no difference in adverse events between two groups except one patient who developed a pulmonary infection in the adalimumab group. The pathology of OA is very heterogeneous, with the degree of synovitis varying among patients. In one study, synovitis was not present in half of the patients with early OA [93]. A study examining the benefits of TNF-α blockers in specific subgroups of patients with higher levels of inflammation is needed.

A randomized, double-blind, placebo-controlled, multicenter study [NCT02471118], subcutaneous injection of adalimumab for knee OA with inflammation is recruiting status in a Canadian [94]. Study designed to evaluate the clinical efficacy and safety of adalimumab versus placebo when used to treat subjects with a diagnosis of knee OA, and with clinical features of inflammation, whose pain persists despite receiving maximum tolerated doses of conventional therapy. A total of 130 subjects will be entered into the study.

#### 4.1.2. IL-1β Signalling Inhibitors

IL-1β is a key pathogenic factor in OA. Diacerein, a small-molecule IL-1β inhibitor, reduces the number of IL-1 receptors, resulting in a reduction in functional IL-1 heterodimer receptor complexes [95]. In a three-year randomised, double-blind, placebo-controlled trial, 507 hip OA patients received either diacerein or placebo daily. Although the pain and functional impairment associated with OA remained unchanged, diacerein significantly reduced joint space narrowing compared with placebo [96]. A Cochrane review of the effect of diacerein in OA concluded that the small benefit derived in terms of joint space narrowing was of questionable clinical relevance and has been observed only in hip OA [97]. In vitro and experimental models showed a reduction in cartilage destruction with IL-1 inhibition by IL-1 receptor antagonists (IL-1Ra) [98]. Three patients with aggressive erosive hand OA with major disability who had failed conventional treatment were treated with 100 mg anakinra (an IL-1β receptor antagonist) daily subcutaneously; at the third month, an improvement in pain was observed, and the NSAIDs were withdrawn [99]. A randomised, double-blind, placebo-controlled trial involving 170 patients with painful knee OA, whose joints were injected with either 50 or 150 mg of anakinra or placebo control, showed no improvement in the WOMAC score or cartilage turnover after 4 weeks [100].

#### 4.1.3. NO Inhibitors

Preclinical studies have shown that iNOS KO mice are resistant to developing OA [101], and pharmacological inhibition of iNOS reduced OA progression and pain in a monosodium iodoacetate (MIA) rodent model of OA [102]. A recent clinical trial investigated the safety and efficacy of a novel irreversible iNOS inhibitor on slowing OA progression in a cohort of overweight and obese patients with knee OA [103]. The drug failed to slow the rate of joint space narrowing over the course of 96 weeks. Withdrawn: The study stopped early, before enrolling its first participant.

### 4.2. Bone Modulators

#### 4.2.1. Bisphosphonates

It has been suggested that the administration of antiresorptive drugs such as bisphosphonates, which are traditionally used to treat osteoporosis, slows the bone remodelling process and could lead to chondroprotection [104]. Zhu et al. showed that early treatment of ovariectomised rats with alendronate significantly attenuated cartilage erosion by inhibiting subchondral bone loss [105]. Strassle et al. demonstrated that when the bisphosphonate zoledronate was administered in a monoiodoacetate model of painful arthritis in rats, it protected against subchondral bone loss, cartilage degradation and, importantly, pain [106]. In a clinical setting, bisphosphonate treatment inhibits bone and cartilage degradation based on an assessment of biochemical markers, although the joint space narrowing observed on X-rays indicated its failure to attenuate the structural deterioration [107]. In a one-year, placebo-controlled trial that included 59 patients with knee OA treated with zoledronic acid 5 mg intravenously as a single infusion, a significant reduction in visual analogue pain scores versus placebo was seen after six months, but not after 12 months; interestingly, a significant reduction in bone marrow lesions was detected with magnetic resonance imaging (MRI) [108]. In a recent meta-analysis of randomised controlled trials that compared bisphosphonate therapy with placebo or conventional medication, Xing et al. assessed the efficacy of bisphosphonates in OA; 15 studies were eligible for analysis and they included 3566 participants (1517 on bisphosphonates) [109]. It was shown that bisphosphonate therapy leads to significant improvements in pain, stiffness and function in OA patients assessed using the WOMAC score. Clodronate is a first-generation, non-amino bisphosphonate, registered in Europe for the treatment of postmenopausal osteoporosis. In a recent study, effects of clodronate in OA patients were investigated [110]. Clodronate increased SOX9 expression, the transcription factor responsible for progenitor stem cells chondrogenic commitment. This study showed that intramuscular 200 mg clodronate weekly injection increased mesenchymal stem cells maturation toward the chondrogenic differentiation. Clodronate also reduced pain VAS score and improved mental and physical performance in patients. In a randomised, double-blind, placebo-controlled trial, 80 symptomatic knee OA patients received either once weekly intraarticular injection of 2 mg clodronate or saline placebo for 4 weeks with 12 weeks of follow-up [111]. The injection of clodronate is associated with significantly greater benefits than placebo in pain VAS, Lequesne index (looking at pain, maximum distance walked and activities of daily living), patient and physician Global Assessment score, WOMAC pain subscale, and acetaminophen requirement. A 6-month randomised pilot trial of 40 patients with active erosive hand OA showed that intramuscular clodronate is effective on pain with a significant reduction in the consumption of anti-inflammatory or analgesic drugs, and improvement of hands function [112]. Reduction of serum Cartilage Oligomeric Matrix Protein (COMP), which bind type I and type II collagen fibers and catalyse fibrillar collagen assembly, was observed after clodronate treatment.

#### 4.2.2. Strontium Ranelate

Strontium ranelate (SR) is an antiosteoporotic drug capable of changing the balance between bone resorption and bone formation, protecting postmenopausal women from spinal and peripheral fractures [113,114]. It has been hypothesised to act on both subchondral bone and cartilage based on the results of in vitro studies [115]. At a dose of 1800 mg/kg/day, SR significantly attenuated cartilage matrix and chondrocyte loss, and decreased chondrocyte apoptosis, in a medial meniscal tear model using Sprague–Dawley rats [116]. Subchondral bone remodelling was also significantly attenuated in the SR-treated group, as shown by the improved microarchitecture and intrinsic mechanical properties. Reginster et al. presented data from a large randomised clinical trial of patients with radiographic and clinical knee OA [115] and showed that SR had a beneficial effect on the radiographic progression of disease based on joint space narrowing after three years of treatment compared with placebo. The effects on pain appeared to be more modest and were only significant for the 2 g group, as assessed using the total WOMAC score and pain subscore. Serial quantitative MRI was analysed in 330 patients to evaluate the effect of SR on cartilage volume loss and bone marrow lesions [117]. The higher-dose of SR (2 g daily) resulted in reduced cartilage volume loss at the tibial plateau versus placebo, assessed after one and three years. In patients with bone marrow lesions in the medial compartment at baseline, a significant decrease in the bone marrow lesion score was detected at 36 months in both treatment groups. These results suggest a beneficial effect of SR on structural progression of primary knee OA.

#### 4.2.3. NGF Inhibitors

Biologic agents that inhibit NGF (fasinumab, tanezumab, and fulranumab) have been tried in OA and have shown promising results in terms of pain relief and improved functional capacity [118,119]. Individuals with knee or hip OA, according to the American College of Rheumatology criteria with radiographic confirmation and who were receiving partial symptom relief with NSAIDs, may benefit more from tanezumab monotherapy; adverse events were more frequent with tanezumab than with NSAIDS, however, and were highest with both in combination [118]. Unfortunately, the rapid progression of OA in NGF inhibitor treated group led the US Food and Drug Administration to impose a partial clinical hold for OA [119]. Three clinical trials are currently being conducted to evaluate the efficacy and long term safety of fasinumab in patients with pain due to osteoarthritis of the knee or hip [NCT03161093] [120] [NCT02683239] [121], and [NCT03304379] [122].

## 5. Conclusions

Until recently, studies of OA have focused mostly on the inflammatory response within the synovium and articular chondrocytes. Currently, the role of periarticular tissues such as subchondral bone and skeletal muscle is gaining recognition and the mechanism of pain generation independent of cartilage degradation is being increasingly pursued. Experiments using more relevant animal OA models and large-scale clinical trials are needed to evaluate the efficacy of various therapeutic targets.
